# 2490: Childhood obesity: A profile of measures of executive functions, emotional processing, and inflammation

**DOI:** 10.1017/cts.2017.113

**Published:** 2018-05-10

**Authors:** Mary S. Rodriguez-Rabassa, Kaumudi Joshipura Jinraj, Maribel Campos Rivera, Vasiliki Michopoulos, Yasuhiro Yamamura

**Affiliations:** University of Puerto Rico – Medical Sciences Campus, San Juan, Puerto Rico

## Abstract

OBJECTIVES/SPECIFIC AIMS: Childhood obesity has become an issue of some concern worldwide. Some reviews and a recent study in adults have indicated that obesity-related inflammatory responses produce brain damage. However, studies exploring associations between inflammation and executive functions in children are overlooked. Therefore, the objective of this cross-sectional study is to determine whether difficulties in executive functions and emotional processing are associated with obesity and inflammation. METHODS/STUDY POPULATION: We have recruited 12 of a total of 60 children aged 6–8 years old. They have completed the NIH Toolbox Cognition Battery and the NEPSY II Affect Recognition tests. Samples of plasma and saliva were collected to quantify inflammatory biomarkers cytokines (IL-6 and TNF-α) assay by Luminex procedure. We performed descriptive analysis and Mann-Whitney *U* test to compare obese Versus nonobese groups. RESULTS/ANTICIPATED RESULTS: Obese children have lower scores in measures of affect recognition than healthy weight children. They also showed higher median scores in both salivary and plasma IL-6 and TNF-α. DISCUSSION/SIGNIFICANCE OF IMPACT: Although no statistical differences were found among groups in either measurement, these preliminary data based on the initial recruitment suggest that children with higher body mass index may have difficulties in emotional processing. More data will be available after completing recruitment to determine if the association between obesity and affect recognition is significant and if it is mediated by inflammation.

